# Integration and Testing of a Three-Axis Accelerometer in a Woven E-Textile Sleeve for Wearable Movement Monitoring

**DOI:** 10.3390/s20185033

**Published:** 2020-09-04

**Authors:** Menglong Li, Russel Torah, Helga Nunes-Matos, Yang Wei, Steve Beeby, John Tudor, Kai Yang

**Affiliations:** 1 School of Electronics and Computer Science, University of Southampton, Southampton SO17 1BJ, UK; rnt@ecs.soton.ac.uk (R.T.); h.p.nunes-matos@soton.ac.uk (H.N.-M.); spb@ecs.soton.ac.uk (S.B.); mjt@ecs.soton.ac.uk (J.T.); ky2e09@soton.ac.uk (K.Y.); 2 School of Science and Technology, Nottingham Trent University, Nottingham NG11 8NS, UK; yang.wei@ntu.ac.uk

**Keywords:** wearable movement sensing, electronic textiles (e-textiles), wearable accelerometer, joint bending angle monitoring

## Abstract

This paper presents a method to integrate and package an accelerometer within a textile to create an electronic textile (e-textile). The smallest commercially available accelerometer sensor (2 mm × 2 mm × 0.95 mm) is used in the e-textile and is fully integrated within the weave structure of the fabric itself, rendering it invisible to the wearer. The e-textile forms the basis of a wearable woven sleeve which is applied to arm and knee joint bending angle measurement. The integrated e-textile based accelerometer sensor system is used to identify activity type, such as walking or running, and count the total number of steps taken. Performance was verified by comparing measurements of specific elbow joint angles over the range of 0° to 180° with those obtained from a commercial bending sensor from Bend Labs and from a custom-built goniometer. The joint bending angles, measured by all three sensors, show good agreement with an error of less than ~1% of reading which provides a high degree of confidence in the e-textile sensor system. Subsequently, knee joint angles were measured experimentally on three subjects with each being tested three times on each of three activities (walking, running and climbing stairs). This allowed the minimum and maximum knee joint angles for each activity to be determined. This data is then used to identify activity type and perform step counting.

## 1. Introduction

Electronic textiles (e-textiles) are becoming widely used in fashion, sports, medical and military applications [1,2]. The global e-textiles market size has grown significantly and is expected to reach $5369 million by 2022 up from $943 million in 2015 [3].

E-textiles play an increasing role in improving healthcare and safety [4,5]. Electronic sensors woven into a textile can track, for example, heart rate, stress and the temperature of the wearer [5,6]. Accelerometers are one of the most common electronic sensors used to detect body motion [7] and other applications of accelerometers include aircraft and missile navigation systems, vibration monitoring, image and rotation stabilisation in digital cameras, and drone flight control [8,9,10]. Recently, researchers have used accelerometers in e-textiles to assess fall risk [11], analyse ambulatory human motion [12], quantify physical activity [13] and monitor body movements during rehabilitation [14].

Singh et al. [15] reports the use of an accelerometer, magnetometer and gyroscope to measure body posture and movement to identify the effect of chair type on work-related musculoskeletal discomfort. The device used was an I2M Motion SXT device with a size of 48.5 mm × 36 mm × 12 mm [16] attached to four body positions (forehead, upper chest and both upper arms). Setuain et al. [17] used a STT-IBS inertial measurement unit (size: 36 mm × 15 mm × 46.5 mm) [16] attached to three locations on the arm to measure the shoulder’s range of motion to monitor patient rehabilitation. Chutatape et al. [18] reported the use of an accelerometer in a smart phone attached to the leg to monitor hip-joint angles. The aim was to avoid improper postures and so reduce the risk of joint dislocation. Chiang et al. [19] reported the use of an accelerometer sensor node (size: 48.5 mm × 36.5 mm × 13.5 mm) to monitor the knee’s range of motion during the recovery progress of degenerative osteoarthritis patients.

The relatively large size of the accelerometer implementations in the above examples is potentially inconvenient and uncomfortable for the wearer [20,21] and may also influence the movement being measured. Large size also presents challenges for accelerometer integration within clothing fabric to achieve a wearable solution [22,23,24]; in the examples above the chosen sensor is not part of the clothing fabric but is attached to the clothing or the person [25]. This can result in a difference between the movement of the sensor and the person, thus impacting on accuracy [26,27]. The sensor may also detach during strenuous exercise. Finally, any fabric-based wearable accelerometer implementation should ensure the movement of the wearable accelerometer is identical to the movement of the person [28,29,30].

This paper presents a miniature accelerometer solution integrated seamlessly within the fabric of a sleeve which closely couples to body movement. A method to integrate and package two microelectromechanical system (MEMS) accelerometer sensors within the textile of a sleeve, via weaving and embroidery, is presented. This e-textile movement sensor sleeve is then used to monitor body motion and identify activity type.

Two of the smallest (at the time of conducting the research) commercially available accelerometers (size: 2 mm × 2 mm × 0.95 mm) were successfully woven into the textile to address the limitations of the state of the art described above. The small size of the accelerometer and the flexible and invisible integration within the textile provides improved user comfort and unobtrusive sensing. The demonstrator performance is then validated for arm movement for comparison before a final demonstration monitoring leg movement. Finally, the data are used to identify the activities of walking, running, climbing stairs and to count the number of steps taken.

Section 2 of this paper describes the e-textile fabrication process. The methods to measure joint bending angle, detect activity type and step count are presented in Section 3. The experimental results and verification are discussed in Section 4. Finally, conclusions are provided in Section 5.

## 2. E-Textile Fabrication

The fabrication of the e-textile is in two stages: The first is to manufacture the electronics, and the second is to integrate the electronics into the textile. The movement sensor specified is an mCube MC6470 device which consists of a three-axis linear accelerometer and a three-axis magnetic field sensor [31]. This device was chosen because it comes in a 2 mm × 2 mm × 0.95 mm 12-pin Land Grid Array (LGA) package and is, the smallest commercially available multi-degree of freedom device of its type. This makes it ideal for unobtrusive integration into an e-textile. The sensor can be programmed with a ±2, 4, 8 and 16 g full-scale acceleration range. In addition, the sensor communicates using the Inter-Integrated Circuit (I^2^C) two-wire serial data protocol thus minimising the amount of wiring required between the device and any central control and communications unit. The magnetic field sensor was not used in this work.

**Figure 1:**
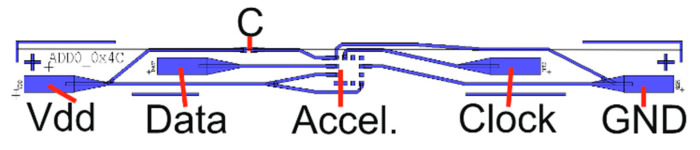
Flexible strip circuit top view with MC6470, 4 external connection pads (Vdd, Data, Clock and GND) and de-coupling capacitor (C). Overall strip dimensions: 40 mm long × 3.3 mm wide.

For this e-textiles implementation, the circuitry is designed to fit on narrow flexible strips made from copper coated polyimide. These filament style circuit layouts allow integration within the weave of the textile, potentially folding into a seam or covering via embroidery. To achieve this, it is essential to make the circuit as narrow as possible (<5 mm wide). To allow a narrow circuit using the MC6470 it was necessary to minimise the number of external pin connections required. By fixing the communication protocol and the device address the number of external connections required was reduced to just 4 wires: I^2^C Data, Clock, Power and Ground. The final circuit was designed using EagleCAD and then exported to L-Edit to create a photomask. The design is shown in Figure 1.

**Figure 2:**
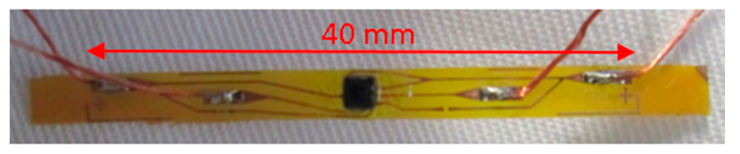
Completed filament circuit photograph with MC6470 and external wires soldered and encapsulated.

The circuit is fabricated using a standard photolithographic process plus a wet copper etch process developed specifically for these e-textile strip circuits and described previously [32,33]. The substrate is a 25 µm thick polyimide laminated with an 18 µm thick copper layer. Once the circuit was etched, the components were soldered to the copper using lead free solder paste (LFS-UFP, BLT Circuits) at 230 °C for 60 s on a hot plate. An additional underfill adhesive (Loctite 4902, Henkel Adhesives) was used to improve the adhesion and peel strength of the components [34]. For the external connections, a multi-strand copper Litz wire of 350 µm total diameter was soldered to the respective pads on the strip. Finally, a glob-top of UV-curable polymer (EC-9519) was dispensed on top of the components and wire joints to provide additional robustness and encapsulation. A completed filament circuit photograph is shown in Figure 2.

**Figure 3:**
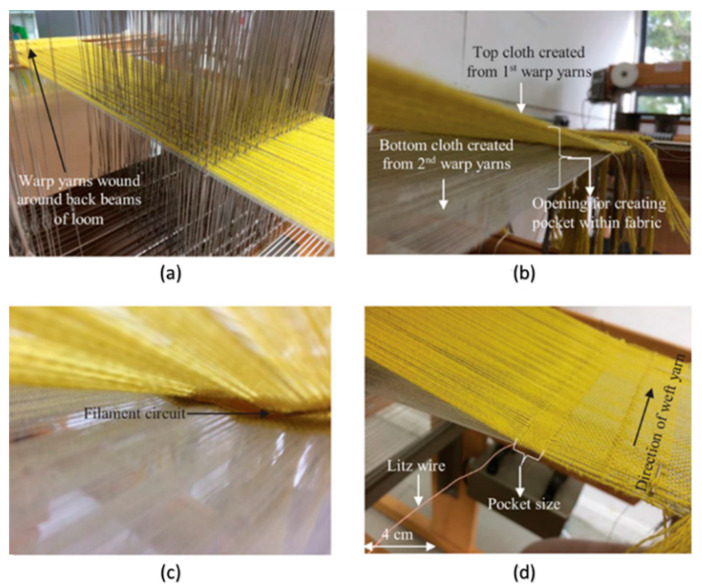
Example weaving process for integrated e-textile fabrication: (**a**) warp yarns on programmable dobby loom, (**b**) weaving of double weave structure, (**c**) insertion of filament circuit and (**d**) closure of pocket and trailing wire.

Having completed the circuit fabrication, the next stage is integration into the textile. A Toika Dobby Weaving Loom was used to weave the circuits into the non-stretchable fabric. The loom has a programmed pattern for the warp yarns (the direction of the final fabric roll) but the shuttle is moved manually for the weft yarns (the direction across the fabric roll). For this work, a cloth was woven in a double weave structure providing pocket structures inside the fabric while it is woven. At the appropriate points in the design, the circuits were inserted into the fabric pockets before continuing with the chosen fibre yarns (a blend of polyester and cotton) to then close the pocket around the circuit. The external wires from the circuit were then allowed to protrude from the ends of the fabric as required in this case; previous results have shown how they can be fully integrated if desired [32]. Example images from the process are shown in Figure 3.

After weaving, the piece of fabric with the integrated accelerometer was stitched on to a Lycra cuff on which hook and loop fastenings were stitched to allow the positioning to be adjusted and improve conformability to the wearer. The wires for the sensors were overstitched using a PFAFF Creative 3.0 embroidery machine. This method was used to gather the wires together into a channel along the edge of the fabric while also allowing the wires a small degree of movement inside the embroidered pattern thus reducing strain on the wires due to cuff rotation and stretching.

**Figure 4:**
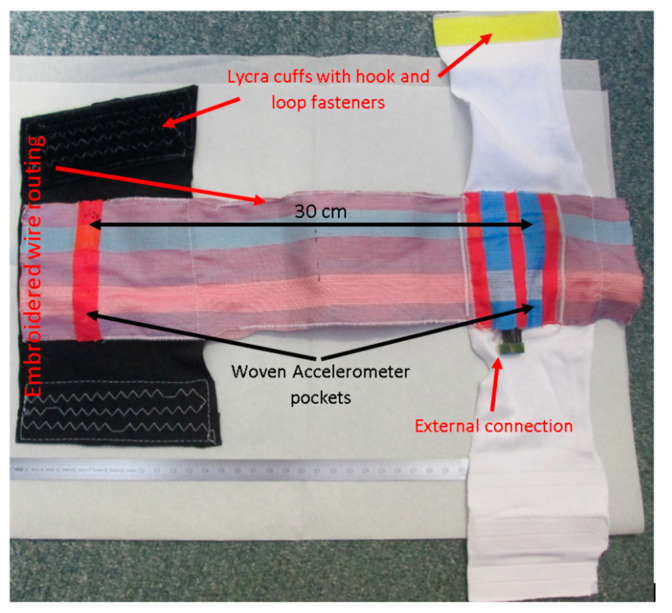
E-textile demonstrator with two woven accelerometers and embroidered track routing, sewn onto Lycra cuffs with hook and loop fasteners.

Two sensors were woven into the fabric for these trials each spaced 15 cm from a central fulcrum (e.g., the elbow or knee). The wires from each sensor were routed on the fabric to meet at a point close to the “top” sensor. A separate Data connection was necessary for each of the two accelerometers, resulting in a total of 5 connections to the e-textile: Data1, Data2, Clock, Power and Ground; thus a 5-pin external connector was used. The connections were then soldered to a standard through hole socket (Amphenol FCI Dubox-304LF) connected via an unshielded lead to an analogue switch (Texas Instruments TS5A63157) and an Arduino Uno microcontroller. The Uno was programmed to switch between the two accelerometers, record their data via I^2^C, and then transmit this via a serial connection to a computer. Although this is relatively bulky approach it was considered acceptable for this prototype system because we have previously demonstrated it is possible to integrate a microcontroller on the flexible polyimide strip using the same fabrication method [32]. The final fabric sleeve with two woven accelerometers and embroidered wire routing is shown in Figure 4.

## 3. Measurement Method

**Figure 5:**
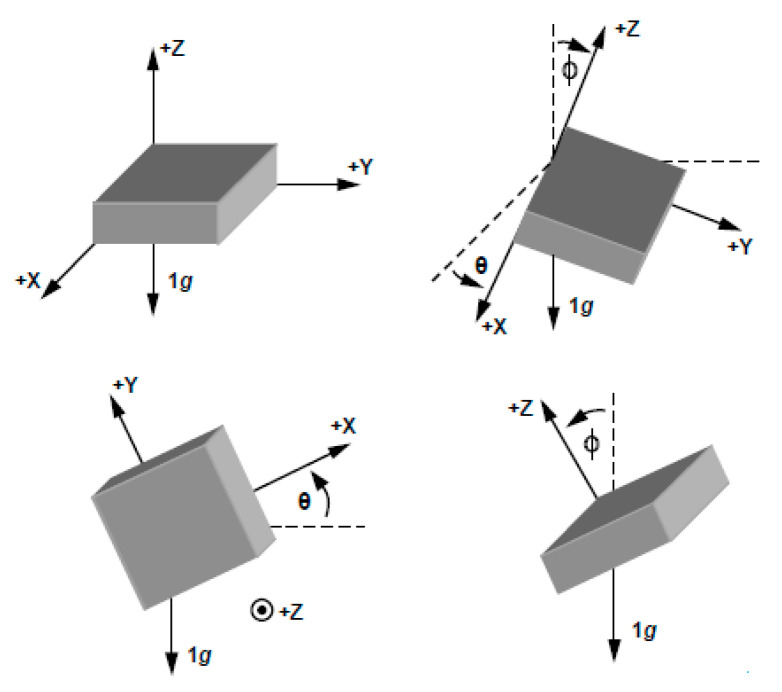
Triple-axis accelerometer to measure angles [35].

The bending angle, activity type and total number of steps were calculated using measurements from the accelerometer. Figure 5 shows an example of accelerometer movement in three dimensions. The mCube accelerometer provides acceleration measurement in three axes and this data can be used to calculate the bending angle based on two axes (termed the inclination angle) or based on three axes (termed the inclination angle and the tilt angle). As accurate miniature three-axis accelerometers are readily available and low cost they provide a convenient method of measuring bending angles in joints.

For a two-axis accelerometer, the method to convert the measured acceleration to an inclination angle is to compute the inverse sine of the *x*-axis and the inverse cosine of the *y* axis as shown in Equation (1) [35],

(1) $$\frac{A_{X,out}}{A_{y,out}}\;=\;\frac{1g\;\cdot \;\;Sin\left(\theta \right)}{1g\;\cdot \;cos\left(\theta \right)}\;=\;\tan\left(\theta \right)$$

where $A_{X}$ is the acceleration in *x* axis, $A_{y}$ is the acceleration in *y* axis and inclination angle *θ* is in radians.

When using a three-axis accelerometer and, if both the tilt and inclination angles are required, the classical method of rectangular (*x*, *y*, *z*) to three-dimensional (3D) spherical conversion can be used to obtain Equations (2) and (3) [35], which allows the calculation of the tilt angle *θ* in the *xy*-plane, and the inclination angle *ϕ* to the gravity vector (the *xz*-plane), from the measured acceleration in each axis.

(2) $$\theta =tan^{-1}\left(\frac{A_{x,out}}{A_{y,out}}\right)$$

(3) $$\emptyset =cos^{-1}\left(\frac{A_{z,out\;}}{\sqrt{A_{x,out}^{2}+A_{y,out}^{2}+A_{z,out}^{2}}}\right)$$

Typically, researchers have adopted two approaches to obtain joint angle. In the first approach, the joint angle is obtained using the orientation of the accelerometer axes with respect to a common fixed reference frame [36]. In the second approach, the joint angle is calculated using both accelerometer and gyroscope readings [36]. In this paper, the first approach is used since only a three-axis accelerometer is required obviating the need for a gyroscope.

**Figure 6:**
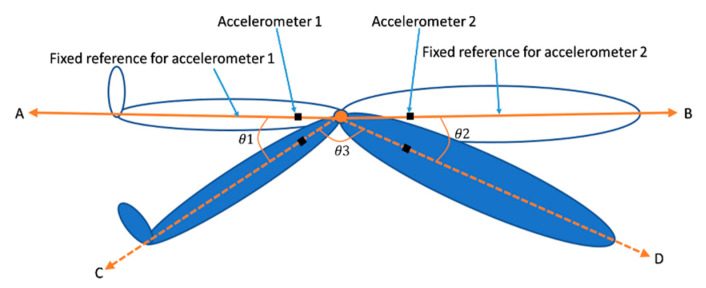
Diagram to show joint bending angle between an upper and lower limb.

As shown in Figure 6, the axes *A* and *B* are the common fixed reference for accelerometers 1 and 2, respectively; axes *C* and *D* are the axes for accelerometers 1 and 2, respectively, after movement; and the angles $\theta_{1}$ and $\theta_{2}$ between the two axes are calculated as shown in Equations (4) and (5), respectively [37]. In this calculation, we assume that the common fixed reference, axes *A* and *B* are in the same plane; therefore, the joint bending angle $\theta_{3}$ is then equal to 180° minus the sum of $\theta_{1}$ and $\theta_{2}$ as shown in Equation (6),

(4) $$\theta_{1}=\arccos(\frac{X_{A}X_{C}+Y_{A}Y_{C}+Z_{A}Z_{C}}{\sqrt{(X_{A}^{2}+Y_{A}^{2}+Z_{A}^{2})(X_{C}^{2}+Y_{C}^{2}+Z_{C}^{2}})})$$

(5) $$\theta_{2}=\arccos(\frac{X_{B}X_{D}+Y_{B}Y_{D}+Z_{B}Z_{D}}{\sqrt{(X_{B}^{2}+Y_{B}^{2}+Z_{B}^{2})(X_{D}^{2}+Y_{D}^{2}+Z_{D}^{2}})})$$

(6) $$\;\;\;\theta_{3}=180{}^{\circ}-\;(\theta_{1}+\theta_{2})$$

where ($X_{A}$ $Y_{A}$ $Z_{A}$) and ($X_{B}$ $Y_{B}$ $Z_{B}$) are the outputs of accelerometers 1 and 2 at a common fixed reference axis, respectively, and ($X_{C}$ $Y_{C}$ $Z_{C}$) and ($X_{D}$ $Y_{D}$ $Z_{D}$) are the readings of accelerometers 1 and 2 after movement to axes *C* and *D*, respectively.

**Figure 7:**
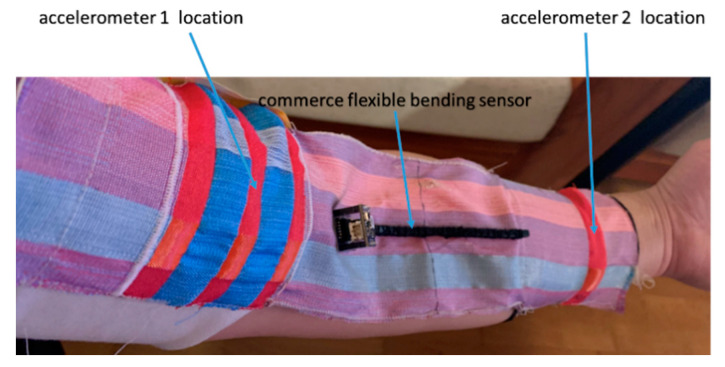
E-textile movement sensor and Bend Labs sensor strapped onto the arm to measure arm joint angle.

To measure the arm’s joint angle, the e-textile movement sensor was tightly strapped on the arm as shown in Figure 7. Accelerometer 1 was located on the palm side of the upper arm, and accelerometer 2 was located on the palm side of the lower arm. The Arduino module was connected via a 0.5 m cable and anchored on a desk to ensure it did not interfere with the movement. For the knee bending angle measurements, the same set-up was used but this time with the e-textile strapped to the leg; both sensors faced forward on the top surface of the thigh and shin.

In this paper we calculate the joint bending angle in the sagittal plane by using the accelerometer data in the *X*, *Y* and *Z* axis to determine activity type, using Equations (4)–(6). The different accelerometer output values depend on their orientation as shown in Figure 5. To calculate the angle in between them on the sagittal plane, the accelerometer data related to movement in the sagittal plane is used whether the wearer is dynamic or static.

As a demonstration of the potential of this e-textile, a study to identify the activity type (such as walking or running) was conducted, using an analysis of knee joint angle to distinguish different activities. de David et al. have compared knee flexion during walking and running; their data indicated a lower knee flexion during running [38]. To determine the difference in knee bending angle between walking, running and climbing stairs, in this paper the knee angle data samples analysed during each activity were obtained from three healthy adult volunteers aged under 50 years old (University of Southampton ethics approval reference number: ERGO/FEPS/57031).

The average knee bending range for each activity is used to calculate the total number of steps of the wearer. For example, assuming the standing knee angle is 180° we count a knee angle change from 180° to 115° and back to 180° as 1 cycle and therefore counting the number of cycles, via MATLAB, equates to the total number of steps. 115° was chosen in this case as an example of full and achievable bending by the subject.

## 4. Results and Verification

The e-textile movement sensors have been used in experiments to detect arm and knee joint angle, identify activity type and count the total steps during an activity. MATLAB (MathWorks Inc., Natick, MA, USA) software was used to analyse the data detected by the accelerometers. The arm joint bending angle has been compared and verified by two other methods. The first method used a direct visual comparison with a bespoke goniometer designed specifically for measuring arm joint bending angle. The second method used a real-time digital comparison with a commercial flexible bending sensor from Bend Labs [28] shown attached to the e-textile in Figure 7. The Bend Labs sensor was chosen because the sensor is wearable, flexible and low-power with sub-degree accuracy [39]. The sensor is recommended by the manufactures for use in joint angle measurements [40] and is straightforward to attach to the fabric providing a reliable digital comparison to the e-textile movement sensor. The two alternative measurement methods enable comparison and verification of the data for the arm joint angle measured by the e-textile.

### 4.1. Arm Joint Angle Measurement and Verification

**Figure 8:**
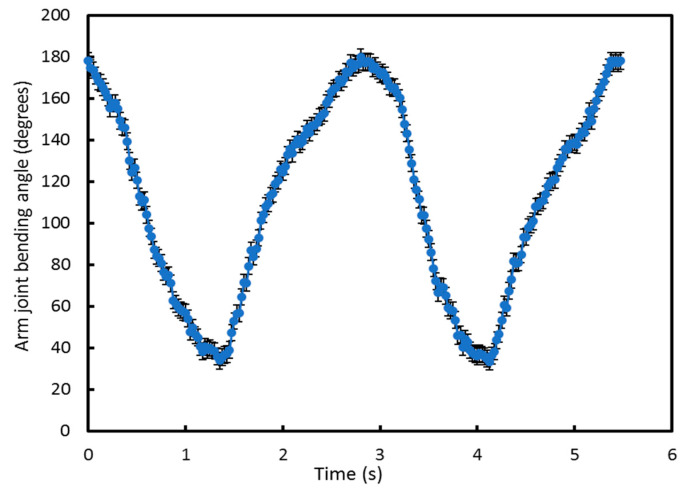
Initial arm joint bending angle measurement results using the e-textile to identify the movement limits.

An initial experiment, performed five times, was used to confirm the basic operation of the e-textile measurement system and that the software could be used to identify the movement cycles. Figure 8 shows the arm joint bending angle calculated in MATLAB from measurements made with the e-textile accelerometers during movement. In this test, the arm was bent from 180° (maximum joint bending limit) to 32° (minimum joint bending limit) and then back to 180°. This is defined as one movement cycle. Using the Arduino module, the accelerometer was set to detect joint angle data at 40 Hz as a suitable compromise of sampling rate, energy consumption and to avoid high electromagnetic interference at the mains power supply frequency of 50 Hz. Figure 8 shows two movement cycles undertaken over 6 s from 180° to 32° and back to 180°. The arm moved naturally during each cycle which was not at a constant speed, explaining why the output graph is not perfectly smooth. As a consequence, the data points appear more densely packed at different points on the graph because the arm speed naturally slows as it approaches the maximum bending angle. However, the sampling rate remains the same throughout. The error bar represents the inaccuracy of the specific accelerometer sensor, typically ±0.5 degrees.

**Figure 9:**
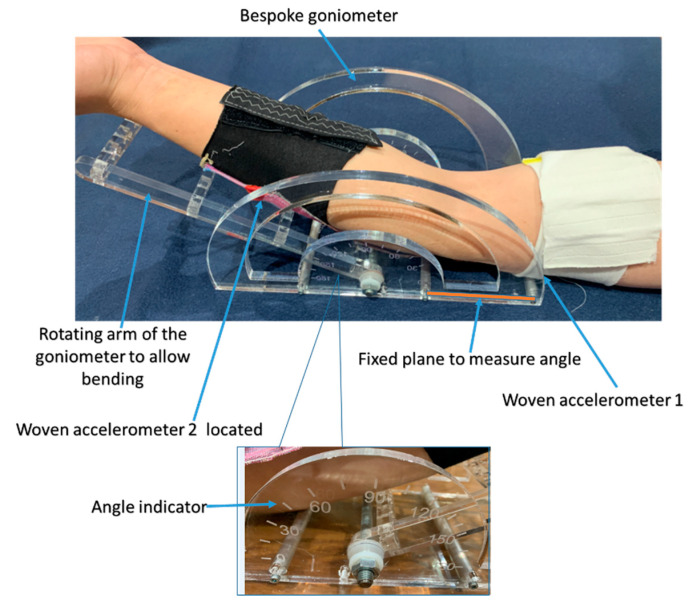
Bespoke goniometer with the e-textile mounted arm to show the verification of joint bending angle.

Having confirmed the basic operation of the e-textile sleeve, the next tests compared the arm joint bending angle measured with the e-textile to that measured with the bespoke goniometer, shown in Figure 9. To compare the e-textile movement sensor with the bespoke goniometer, accelerometers 1 and 2 were located on the upper and lower arm, respectively, but for this test the e-textile is worn on the back of the arm, positioned with one accelerometer on the triceps and the other on the forearm. Placing the e-textile sensors on the back of the arm ensures they are as close as possible to the goniometer, thus improving the verification accuracy. The triceps sensor rested against a fixed planar surface whilst the forearm moved along with the goniometer arm.

The goniometer arm is free to move from 0° to 180° and has scale markings at 0°, 15°, 30°, 45°, 60°, 75°, 90°, 105°, 120°, 135°, 150°, 165° and 180°. In this verification trial, only the angles from 45° to 180° on the scale were measured because 180° is the maximum joint bending limit and 32° is approximately the minimum joint bending limit for the arm but it is participant-dependant, so 45^o^ was chosen so that all participants could easily achieve it without discomfort. The accelerometer is programmed to record the bending angle every 6 s, giving sufficient time for the rotation arm of the goniometer to be moved to the next angle. In this verification test, the forearm (accelerometer 2) rests on the rotating arm of the goniometer and the triceps (accelerometer 1) rests on the fixed horizontal plane of the goniometer and does not move. The rotating arm was moved from 180° to 45° to match the scale graduations on the goniometer, as shown in Figure 9.

Table 1 shows the mean value of the accelerometer reading at each angle of the goniometer calculated from five readings at each goniometer angle. The accelerometer derived angles show strong agreement with the corresponding goniometer position. The small error (~1% of reading) is due to a combination of user measurement error from both the accelerometer output and the positional accuracy of the goniometer along with positional offset between the accelerometers and the goniometer axes.

A two-axis commercial flexible bending sensor from Bend Labs was then used to provide an additional verification of the accelerometer-based sensor results. The bending sensor is attached across the central fabric section of the movement sensing sleeve, as shown in Figure 7. The two accelerometers and the commercial flexible bending sensor are aligned to the back of the arm for this test. The bending sensor output is transmitted via the built-in Bluetooth transmitter to an Android phone (MEIZU PRO 7 Plus) running Bend Labs own bespoke SensorDemo software [35]. In this verification test, four angular positions (180°, 135°, 90° and 45°) were measured by the e-textile sensor sleeve and the Bend Labs sensor.

Table 2 shows the Bend Labs and e-textile movement sensor measurement results at the four fixed angle positions which were verified using the goniometer. The e-textile and Bend Labs sensor’s measured angles show good agreement with each other and the goniometer at each of the four fixed angle positions. The maximum error is less than ~1% of reading.

This validation experiment confirms that the new e-textile system provides a sufficiently accurate output for joint angle measurement. This, therefore, gives confidence for the further experimental tests concerning measurement of knee joint angle, identification of activity type and step counting.

### 4.2. Knee Joint Angle Measurement

The e-textile sensor was strapped to the knee (using the same method as the arm, shown in Figure 7 to measure the knee joint bending angle; accelerometers 1 and 2 were located onto the inner side of the leg and onto the lower thigh and upper calf, respectively. The e-textile movement sensor needs to be sufficiently tight against the leg to avoid measurement inaccuracies that would occur from a loosely fitting system. A loose-fitting textile would allow the accelerometers to move independently from the limb and therefore any output would no longer relate to the angle of a particular joint. The e-textile sensor was set to measure the accelerometer data at 40 Hz as before.

**Figure 10:**
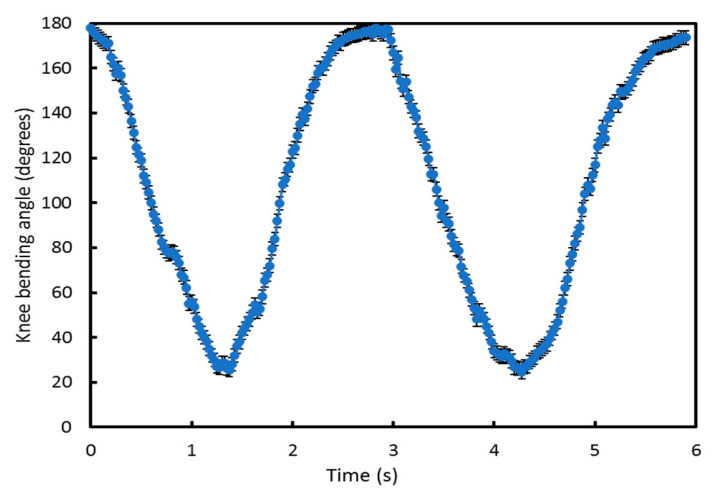
Knee joint bending angle measured by the e-textile movement sensor.

Figure 10 shows the results of an initial output verification trial of two cycles of knee joint bending used to demonstrate the system is working, that the knee angle can be measured and the boundaries of this movement. This test started with the subject standing (knee joint bending angle is 180°), then the subject bent down to the minimum knee bending angle they could achieve and then returned back to 180°. The results in Figure 10 show that the minimum knee bending angle is 25° and also shows the rate of change in angle as the knees bend from 180° to 25° and return back to 180°. Comparing this with Figure 8 shows different rates of change of angle for the arm and knee during testing.

### 4.3. Identification of Activity Type and Step Count during an Activity

**Figure 11:**
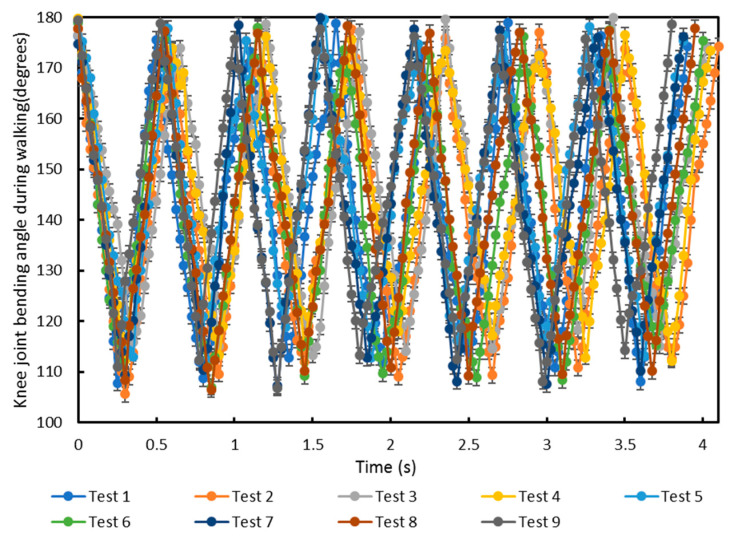
Knee joint bending angle for nine tests monitored using the e-textile movement sensors whilst walking. Tests 1, 2 and 3 were on subject 1; tests 4, 5 and 6 were on subject 2; and tests 7, 8 and 9 were on subject 3.

For different activities, the knee joint bending angle will have different ranges [41]. To identify the minimum and maximum knee bending angle for each activity, measurements were made on three healthy subjects when walking, running and climbing stairs with the same 40 Hz accelerometer sampling rate. Each subject was tested on a different day. In the test, the subject’s knee bending angle was firstly measured when walking, running and climbing stairs. Then, the system was switched off and after a 20 min delay, the system was switched on again and the subject retested. Each subject was tested three times on a single day. The knee bending angle for seven walking steps by the three subjects is shown in Figure 11. Each subject walked at a slightly different speed and stride length. This caused the average step frequency to be different but the maximum and minimum knee joint angles in Figure 11 show close agreement. The minimum and maximum range of knee bending angles for the nine tests during walking are 105° to 115° and 170° to 180°, respectively.

For completeness, the equivalent graphs for knee joint bending angle whilst running and climbing stairs are included in Appendix A.1 and Appendix A.2. Table 3 shows the minimum and maximum range of knee bending angles for the three subjects during walking, running and climbing stairs. Therefore, the unique minimum and maximum angles can be used to distinguish the activity type (e.g., if walking, a minimum of 105° to 115° and a maximum of 170° to 180°). 

**Figure 12:**
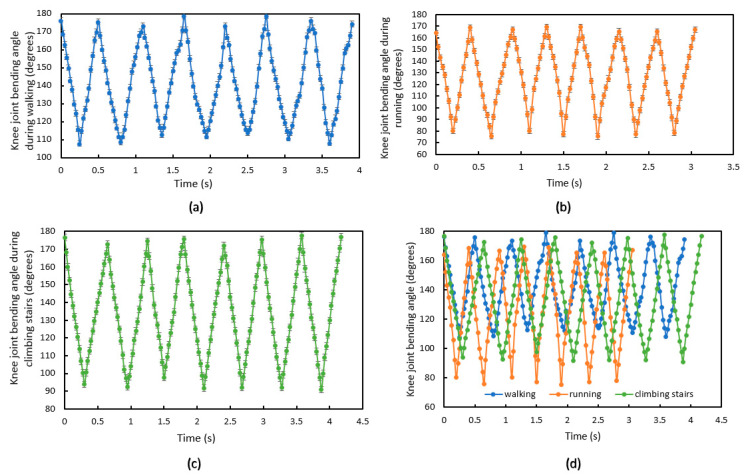
Knee joint bending angle for subject 1 using the e-textile movement sensors while (**a**) walking; (**b**) running; (**c**) climbing stairs; or (**d**) walking, running and claiming stairs combined.

Compared to the results from walking, when running the knee bends around 35° more, which means the knee joint needs to withstand more bending force during running. The test results for climbing stairs shows that the knee bends ~15° more than walking and ~20° less than running, which means the knee joint needs to withstand more bending force than walking but less bending force than running. Figure 12a–c shows a single subject’s knee bending angle for seven steps when walking, running and climbing stairs, respectively, to further show knee bending angle differences. Figure 12d shows the three measurements combined on a single graph. Running shows the highest step frequency.

## 5. Conclusions

The presented fabrication process allows a small integrated strip circuit to be fabricated, and this highly flexible strip circuit contains the smallest commercially available 3-axis accelerometer (2 mm × 2 mm × 0.95 mm). Weaving a flexible electronic strip into a fabric provides more convenience and comfort for the wearer, when compared with previously reported methods [15,16,17,18,19].

The fabric with integrated miniature 3-axis accelerometer has been applied to measure limb movement angle with post processing of the accelerometer data being performed in MATLAB. The angles measured by the e-textile movement sensor have been verified using a goniometer and a commercial 2-axis bending sensor. The basic accelerometer error is ±0.5 degrees and, when integrated in the textile, a maximum error of ~1% of reading was observed.

The e-textile movement sensor was also used to detect elbow and knee joint bending angle, distinguish activity type (walking, running and climbing stairs) and to count steps. Knee bending range values have been identified to allow the activity type to be distinguished.

This integration demonstrates a platform technology offering the combination of discrete sensing, bendability and comfort for future e-textile applications where movement monitoring is desired. The results were obtained from three people as proof of concept of the e-textile integrated accelerometer for knee movement monitoring. The approach shows significant promise in both the fabrication methodology, and the sensing results, providing a viable solution for movement monitoring which is unobtrusive and comfortable for the wearer, allowing a wide range of activity types to be identified.

## Figures and Tables

**Table 1:** Comparison of the e-textile movement sensor’s measured angles and the goniometer position.

Angles of Bespoke Goniometer	180°	165°	150°	135°	120°	105°	90°	75°	60°	45°
The mean value of angle measured by the e-textile accelerometer calculated from five readings at each goniometer angle	179.5°	165.3°	149.6°	134.7°	120.2°	104.7°	89.8°	75.3°	60.4°	44.6°
Standard deviation	0.32°	0.49°	0.45°	0.28°	0.54°	0.47°	0.38°	0.42°	0.29°	0.31°

**Table 2:** Arm joint bending angle measured by e-textile sensor and Bend Labs bending sensor.

Fixed Position Measured by Bespoke Goniometer	180°	135°	90°	45°
The mean value of angle measured by the e-textile accelerometer calculated from five readings at each goniometer angle	180.3°	134.7°	90.4°	45.3°
Standard deviation for the e-textile sensor	0.43°	0.37°	0.51°	0.39°
The mean value of angle measured by the Bend Labs sensor calculated from five readings at each goniometer angle	178.8°	134.1°	88.3°	44.2°
Standard deviation for the Bend Labs sensor	0.77°	1.12°	0.84°	0.71°

**Table 3:** The maximum and minimum knee bending angle during walking, running and climbing stairs, measured by the e-textile movement sensor.

Activity	Minimum Knee Angle (Degrees)	Maximum Knee Angle (Degrees)
Walking	105°–115°	170°–180°
Running	70°–80°	160°–170°
Climbing stairs	90°–100°	170°–180°

**Figure A1:**
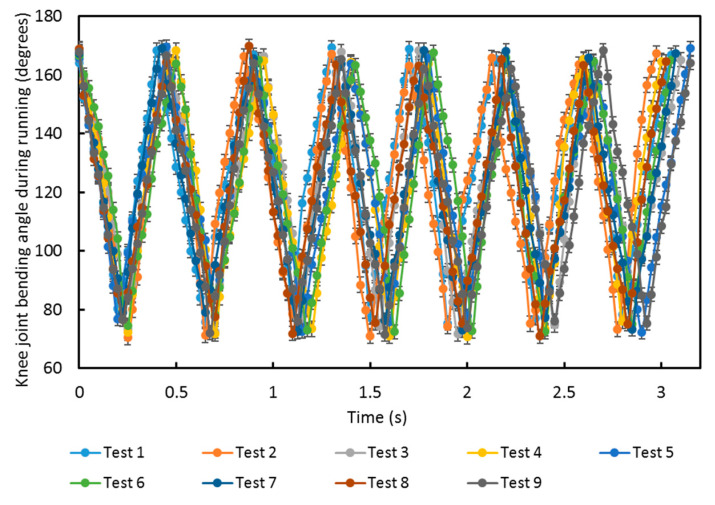
Knee joint bending angle for nine tests monitored using the e-textile movement sensors whilst running. Tests 1, 2 and 3 were on subject 1; tests 4, 5 and 6 were on subject 2; and tests 7, 8 and 9 were on subject 3.

**Figure A2:**
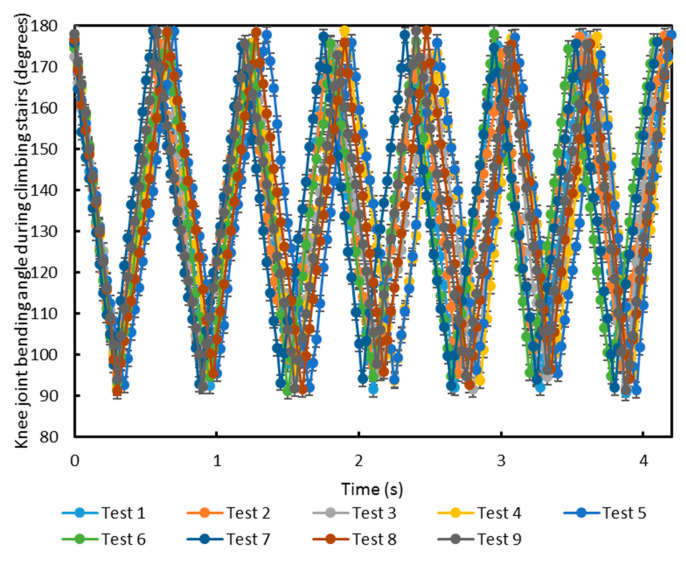
Knee joint bending angle for nine tests monitored using the e-textile movement sensors while climbing stairs. Tests 1, 2 and 3 were on subject 1; tests 4, 5 and 6 were on subject 2; and tests 7, 8 and 9 were on subject 3.
